# Editorial Special Section on Biomedical Diffuse Optics for the Brain

**DOI:** 10.1109/OJEMB.2023.3273048

**Published:** 2023-05-22

**Authors:** Sergio Fantini

**Affiliations:** Department of Biomedical EngineeringTufts University 1810 Medford MA 02155 USA

## Abstract

This special section collects four articles on the application of diffuse optics to measure cerebral hemodynamics and oxygenation. The possibility of using near-infrared light to collect cerebral hemodynamic and metabolic information through the intact scalp and skull was first proposed in the 1970s [1]. Commercial cerebral oximeters were developed in the 1990s, and functional measurements of brain activation, which signaled the birth of functional near-infrared spectroscopy (fNIRS), were first reported in 1993 [2], [3], [4], [5]. Oscillatory cerebral hemodynamics were also investigated in relation to functional and diagnostic applications [6], [7], [8], [9]. Journal special issues were published to celebrate the 20th [10] and 30th [11] anniversaries of fNIRS, and numerous review articles have provided overviews of the field of noninvasive optical measurements of the brain [12], [13], [14], [15].

This special section collects four articles on the application of diffuse optics to measure cerebral hemodynamics and oxygenation. The possibility of using near-infrared light to collect cerebral hemodynamic and metabolic information through the intact scalp and skull was first proposed in the 1970s [1]. Commercial cerebral oximeters were developed in the 1990s, and functional measurements of brain activation, which signaled the birth of functional near-infrared spectroscopy (fNIRS), were first reported in 1993 [2], [3], [4], [5]. Oscillatory cerebral hemodynamics were also investigated in relation to functional and diagnostic applications [6], [7], [8], [9]. Journal special issues were published to celebrate the 20th [10] and 30th [11] anniversaries of fNIRS, and numerous review articles have provided overviews of the field of noninvasive optical measurements of the brain [12], [13], [14], [15].

The four articles in this special issue cover the topics of cerebral oximetry, fNIRS, and cerebral hemodynamic oscillations.

Nguyen et al. describe a continuous-wave (CW) method to perform absolute measurements of cerebral tissue oxygenation using a single source-detector distance [A1]. Absolute measurements of optical properties of tissue typically require time-resolved methods (either in the time domain or in the frequency domain) and/or data collection at multiple source-detector distances. In this contribution, Nguyen et al. measure an absolute tissue saturation from CW data collected at a single source-detector distance (either 30 mm or 40 mm), and at three wavelengths (730, 800, and 850 nm), after making some assumptions about scattering properties and differential pathlength factors (which depend on both absorption and scattering properties of tissue) at the three wavelengths. Despite the need for a number of assumptions, the ability of performing cerebral oximetry with CW optical data collected at a single source-detector distance has important practical implications for the development of miniaturized, wearable, and cost-effective instrumentation.

Dale et al. report an approach to motor-task classification based on high-density frequency-domain fNIRS and a 3D convolutional neural network (CNN) [A2]. The emphasis is on leveraging both temporal and spatial features of measured cerebral hemodynamics to classify brain activation using convolutional neural networks (CNNs). This capability extends beyond motor task classification, which is specifically considered in this contribution, as one can conceive applications toward cognitive or mental states classification (sense of urgency, mind wandering, distraction, cognitive load, interference). This is an important step toward an objective assessment and classification of brain activation, which can advance human-computer and brain-computer interfaces, especially if this classification can be performed continuously in real-time.

Yang et al. explore the ability to assess cerebrovascular impedance (CVI) from measurements of oscillatory cerebral blood volume (with CW-NIRS) and blood flow [with diffuse correlation spectroscopy (DCS)] at the cardiac frequency in non-human primates [A3]. During controlled modulations of cerebral perfusion pressure (CPP), the proposed method for impedance measurements based on the ratio of blood volume to blood pressure oscillation amplitudes at the cardiac frequency demonstrates the different dependence of CVI on CPP within and outside the CPP range of intact autoregulation. Achieving a non-invasive measurement of cerebrovascular reactivity, impedance, and autoregulation is highly significant for the assessment of cerebrovascular health. This can have important implications for diagnostics of cerebrovascular pathologies, monitoring therapeutic efficacy or recovery from acute illness, assessing healthy aging or development of chronic disease, etc. While the proposed method in this contribution relies on modulation of CPP and only generates relative changes of CVI, thus resulting in practical limitations to its applicability, it is nevertheless an important step toward the development of non-invasive optical methods for cerebrovascular health assessment.

Fernandez et al. investigate spontaneous cerebral hemodynamics, as measured with CW-NIRS in 78 healthy human subjects, and quantify their coherence with arterial blood pressure (ABP) in the frequency range 0.01–1.0 Hz [A4]. The motivation for this work is the identification of cerebral hemodynamics that are coherent with ABP in an effort to quantify local cerebrovascular microcirculation responses to systemic ABP changes. This can have implications for coherent hemodynamics spectroscopy (CHS) [9] performed without the need of external modulation of ABP. The maximum coherence between spontaneous cerebral hemodynamics and ABP was observed at a frequency of about 0.1 Hz, which is relevant for cerebral autoregulation assessment because intact autoregulation is able to minimize cerebral blood flow changes in response to ABP oscillations at this frequency. Amplitude and phase measurements of oscillations of oxy- and deoxyhemoglobin concentrations that are coherent with ABP (or other driving functional or physiological processes) carry valuable information on local dynamic responses of brain tissue at the microcirculation level. The ability to quantify these local dynamic responses and relate them to functional, metabolic, or pathologic conditions can have important implications for non-invasive optical sensing of the human brain.

In summary, this special issue provides a snapshot of relevant applications of diffuse optics for cerebral oximetry, functional brain assessment, and characterization of cerebral hemodynamic oscillations at the cardiac frequency (around 1 Hz) or at low frequencies (around 0.1 Hz). Non-invasive optical sensing of the brain is an active research field. It has grown and expanded from initial efforts toward understanding light propagation in highly scattering tissue and developing early instruments for diffuse optics (in continuous wave, time domain, and frequency domain), to more recent developments toward commercial translation, clinical applications, and research in a variety of areas such as psychology, sports medicine, neonatology, nutrition, brain-computer and human-computer interfaces, hyperscanning (multi-subject measurements), neuroeconomics, etc. It will be exciting to witness the next breakthrough development in this field.


Sergio Fantini, Area Editor, Biophotonics
Professor and Interim Chair
Department of Biomedical Engineering
Tufts University
Medford, MA 02155 USA
sergio.fantini@tufts.edu


## Appendix Related Articles

[A1] T. Nguyen, S. Park, B. Hill, and A. H. Gandjbakhche, “Single source-detector separation approach to calculate tissue oxygen saturation using continuous wave near-infrared spectroscopy,” *IEEE Open J. Eng. Med. Biol.*, vol. 4, pp. 79–84, 2023, doi: 10.1109/OJEMB.2023.3246929.
[A2] R. Dale, T. D. O'Sullivan, S. Howard, F. Orihuela-Espina, and H. Dehghani, “System derived spatial-temporal CNN for high-density fNIRS BCI,” *IEEE Open J. Eng. Med. Biol.*, vol. 4, pp. 85–95, 2023, doi: 10.1109/OJEMB.2023.3248492.
[A3] J. Yang et al., “Cerebrovascular impedance as a function of cerebral perfusion pressure,” *IEEE Open J. Eng. Med. Biol.*, vol. 4, pp. 96–101, 2023, doi: 10.1109/OJEMB.2023.3236267.
[A4] C. Fernandez et al., “Coherent spontaneous hemodynamics in the human brain,” *IEEE Open J. Eng. Med. Biol.*, vol. 4, pp. 102–108, 2023, doi: 10.1109/OJEMB.2023.3234012.
